# Identifying Individuals with Antisocial Personality Disorder Using Resting-State fMRI

**DOI:** 10.1371/journal.pone.0060652

**Published:** 2013-04-12

**Authors:** Yan Tang, Weixiong Jiang, Jian Liao, Wei Wang, Aijing Luo

**Affiliations:** 1 Department of Radiology, The Third Xiangya Hospital, Central South University, Changsha, Hunan, China; 2 Biomedical Engineering Laboratory, School of Geosciences and Info-Physics, Central South University, Changsha, Hunan, China; 3 Department of Information science and Engineering, Hunan First Normal University, Changsha, Hunan, China; 4 Key Laboratory of Medical Information Research (Central South University), College of Hunan Province, Changsha, Hunan, China; Hangzhou Normal University, China

## Abstract

Antisocial personality disorder (ASPD) is closely connected to criminal behavior. A better understanding of functional connectivity in the brains of ASPD patients will help to explain abnormal behavioral syndromes and to perform objective diagnoses of ASPD. In this study we designed an exploratory data-driven classifier based on machine learning to investigate changes in functional connectivity in the brains of patients with ASPD using resting state functional magnetic resonance imaging (fMRI) data in 32 subjects with ASPD and 35 controls. The results showed that the classifier achieved satisfactory performance (86.57% accuracy, 77.14% sensitivity and 96.88% specificity) and could extract stabile information regarding functional connectivity that could be used to discriminate ASPD individuals from normal controls. More importantly, we found that the greatest change in the ASPD subjects was uncoupling between the default mode network and the attention network. Moreover, the precuneus, superior parietal gyrus and cerebellum exhibited high discriminative power in classification. A voxel-based morphometry analysis was performed and showed that the gray matter volumes in the parietal lobule and white matter volumes in the precuneus were abnormal in ASPD compared to controls. To our knowledge, this study was the first to use resting-state fMRI to identify abnormal functional connectivity in ASPD patients. These results not only demonstrated good performance of the proposed classifier, which can be used to improve the diagnosis of ASPD, but also elucidate the pathological mechanism of ASPD from a resting-state functional integration viewpoint.

## Introduction

It is increasingly acknowledged that there is a very close link between ASPD and criminal behavior, and the study of functional brain connections in ASPD has important implications in a legal context. Recently, resting-state functional magnetic resonance imaging (rs-fMRI) has attracted increasing attention as a method for mapping large-scale neural network function and dysfunction. Based on rs-fMRI, a growing body of studies has focused on the quantitative analysis of the brains of patients with neurological and psychiatric disorders, including depression [1]–[3], Alzheimer’s disease and dementia [4], [5], schizophrenia [6]–[9]. However, to our knowledge, there have been no such imaging studies of people with ASPD. The functional pathological mechanisms of this complex mental disorder remain unclear.

In recent years, many advances have been made using univariable and group-level statistical methods with functional magnetic resonance imaging (fMRI) data. However, these methods are less helpful in the diagnosis of psychiatric disorders [6]. Therefore, increasing attention is being paid to the application of multivariate pattern recognition methods for brain image analysis. Many sophisticated multivariate pattern methods, such as LLE, PCA, SVM, and C-means clustering, have been proposed. Recently, many studies have considered the classification features of resting-state brain functional data, and have successfully discriminated subjects with brain disorders from normal controls. Zen et al. identified subjects with major depression using a SVM classifier [1], Shen et al. developed a LLE+ C-means classifier to discriminate schizophrenics from health subjects [6], and Nico et al. also used a SVM classifier to predict individual brain maturity [10]. These studies consistently showed that a machine learning-based classifier could be used to capture significant neuroimaging-based biomarkers and label new samples [11]. Therefore, it is proposed that such techniques can be used in clinical diagnosis. However, ASPD cohorts belong to a special group of patients. Compared to individuals with other brain disorders, e.g., AD, MCI and schizophrenia, ASPD individuals show fewer structural abnormalities. Accordingly, it may be more difficult to identify ASPD individual than individuals with other brain disorders. Moreover, due to the high dimensionality of fMRI data, noisy measurements and the small number of available training samples, it remains a challenge to abstract sufficient information to classify ASPD from fMRI scans.

Therefore, one purpose of this study was to design an exploratory data-driven classifier that can be used to identify ASPD individuals from controls using rs-fMRI data. Another purpose of the study, building on the first, was to illuminate the abnormal resting-state functional connectivity patterns of ASPD.

## Materials and Methods

### 1. Ethics Statement

All of the subjects in this study were of legal age to give consent (age >18 years old) to participate in the experiment, but they were under the legal age when they entered into the School for Youth Offenders of Hunan Province. Written informed consent was obtained from all the subjects after they were given a detailed description of the study. See Text S1 for details. We confirmed that all potential participants who declined to participate were not disadvantaged in any way by not participating in the study. The participants were paid a base rate of ¥100 for their participation, plus an additional ¥50 bonus according to their performance. This study was approved by the Ethical Committee of the Third Xiangya Hospital of Central South University and the School for Youth Offenders of Hunan Province.

### 2. Participants

Four hundred and eighty volunteers were recruited from the School for Youth Offenders of Hunan Province. All of the volunteers received reformatory education at this school in response to misdemeanor crimes. All of the young offenders have regular school hours every day and “enclosed-style” management is implemented. All of the subjects were of legal age to give consent (age >18 years old) to participate in the experiment, but they were under the legal age when they entered the school. The volunteers were tested in groups at the school using the Personality Diagnostic Questionaire-4+ (PDQ-4+) by a professional with experience in psychological testing. Of these, 122 subjects had ASPD scores, equal to or above 4 score, and were retained for the experiment. The 122 subjects continued to be tested using the Personality Disorder Interview(PDI-IV) [12] by two senior psychiatrists, and 32 subjects were diagnosed with ASPD. The inter-rater reliability for the PDI-IV ranged from a low of 0.57 (narcissistic) to a high of 0.92 (dependent) with a median of 0.83 [13].

Finally, our subjects were 32 ASPD individuals who met the ASPD criteria of the PDQ-4+ and DSM–IV [14] and 35 controls without ASPD who were matched to the ASPD subjects in age and education (Table 1). All the subjects had been sentenced to reformatory education at this school for three years, and their misdemeanors included 42 thefts, 20 cased of swindling, and 6 robberies. There were no political offenses. The subjects were right-handed native male Chinese speakers and had no access to alcohol or illicit drugs for at least 6 months prior to the study. None of the subjects had major head trauma, or a history or current diagnosis of serious mental disorders, e.g., depression, anxiety neurosis or schizophrenia. Each of the subjects was companied by three teachers when they underwent the fMRI scans.

**Table 1:** Characteristics of the participants in this study.

	ASPD	Controls
	(Mean±SD)	(Mean±SD)
Age	20.5±1.37	21.67±2.54
Years of education	8.15±1.54	9.73±0.82
IQ	106.66±12.90	106.84±16.6

ASPD: offenders with antisocial personality disorder.

### 3. Data Acquisition and Preprocessing

During the experiments, the subjects were instructed to keep their eyes closed, relax, remain awake and refrain from performing specific cognitive exercises. The functional MRI images were acquired on a 1.5 T scanner (Avanto system, Siemens). Each functional resting-state session lasted 5 min, and 150 volumes were obtained. The structural data were acquired by T1-weighted brain MRI scans using a standard sagittal 3D MP-RAGE sequence (TR = 2400 ms, TE = 3.61 ms, FA = 8°, FOV = 240×240 mm, slice thickness = 1.2 mm, slices = 160, voxel size = 1×1×1 mm).

The rs-fMRI images were pre-processed using SPM8 (www.fil.ion.ucl.ac.uk/spm). For each subject, the first five volumes of the scanned data were discarded due to magnetic saturation. The remaining volumes were corrected via registering and re-slicing to control for head motion. All subjects in this study had less than 1 mm translation in the x, y, or z-axes and less than 1 degree of rotation in each axis. Next, the volumes were normalized to a standard echo planar imaging template in the Montreal Neurological Institute (MNI) space. Then, smoothing and filtering were performed using a Gaussian filter of 8 mm full-width half-maximum kernel and a Chebyshev band-pass filter (0.01–0.08 Hz) respectively. The processed images were divided into 116 regions according to the automated anatomical labeling (AAL) atlas [15]. Regional mean time series were obtained for each subject by averaging the fMRI time series over all the voxels in each of the 116 regions [16]. Considering several potential sources of physiological noise in the functional connectivity analysis, nuisance covariates including head motion parameters, global mean signals, white matter signals and cerebrospinal fluid signals were regressed out from the image [17]–[19]. Pearson’s correlation coefficients were used to evaluate functional connectivity between each pair of regions and we obtained a resting-state functional network that was expressed as a 116×116 symmetrical matrix for each subject. By removing the 116 diagonal elements, the 6670 upper triangular elements of the functional connectivity matrix were normalized using Fisher’s z-transform, and were then used as the features in the subsequent multivariate pattern analyses.

### 4. Development of Multivariate Pattern Classifier

**Figure 1:**
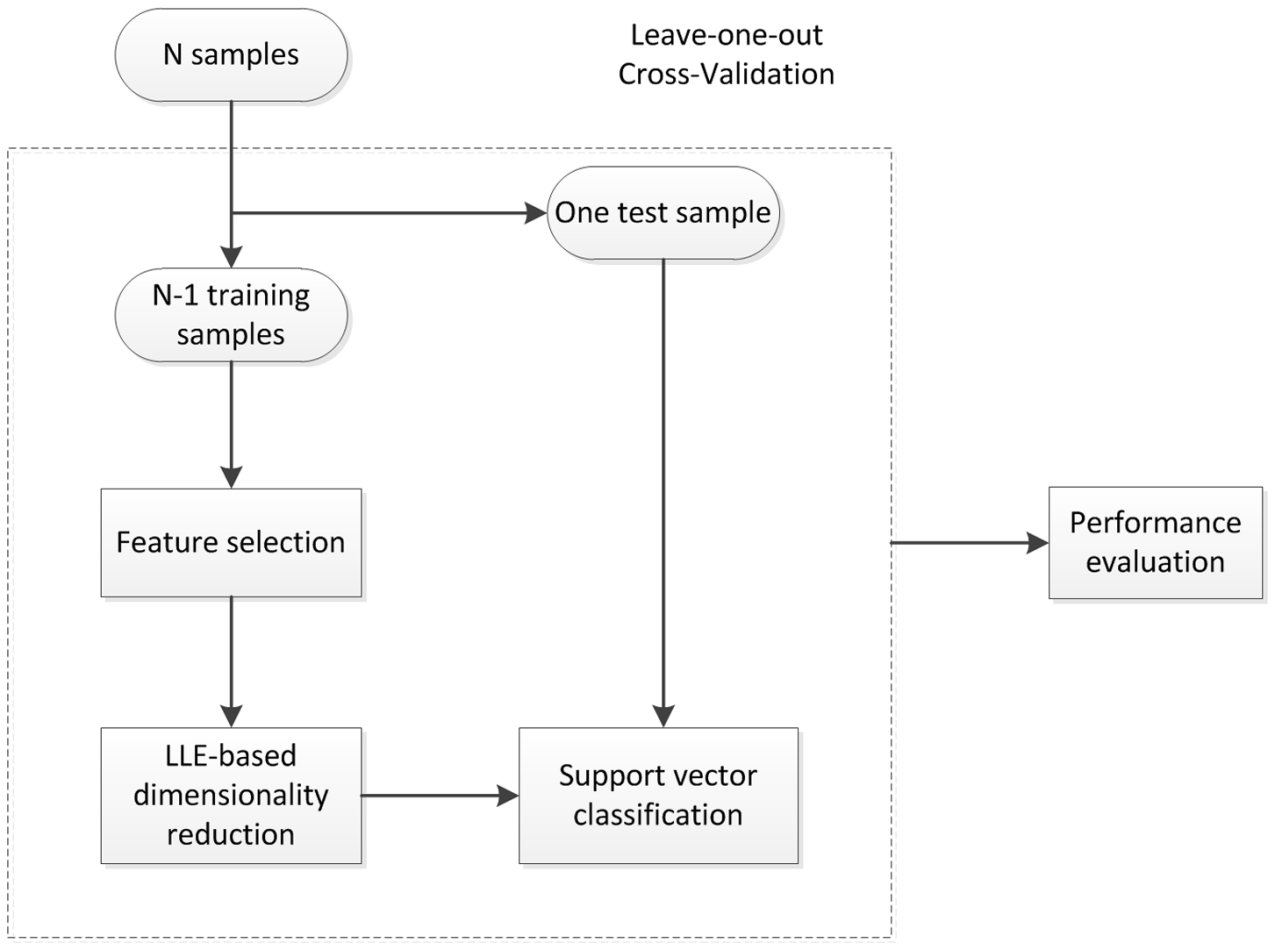
Flow chart of the LDA+SVM classifier.

To discriminate ASPD subjects from matched controls, we developed a data-driven classifier that incorporated four steps: feature selection, LLE-based dimensionality reduction, support vector classification and performance evaluation (Figure 1).

#### 4.1 Feature selection

Feature selection can accelerate computation and diminish noise [6], [10]. It has been verified that through the relevance of a feature to classification, the identifiable power of the feature can be quantitatively measured [20]. In this study, we used the Kendall tau rank correlation coefficient [1], [21] to select a feature subset. The Kendall tau rank correlation coefficient 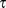 provides a distribution-free test of independence between two variables and measures the relevance of each feature of classification [6]. See Text S2 for details of Kendall tau rank.

In our study, a positive correlation coefficient 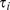 represents a decrease in the *i*th functional connectivity in the ASPD group compared to the control group, and vice versa (+1 for controls and −1 for ASPD). Moreover, this difference increases substantially when the absolute value of the Kendall correlation coefficient 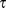 is larger. The absolute value of 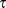 symbolizes discriminative power. We ranked every Kendall correlation coefficient 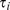 according to its discriminative powers and selected those over a certain threshold as the final feature set for classification.

#### 4.2 LLE-based dimensionality reduction

In this study, we used LLE-based dimensionality reduction. As an unsupervised nonlinear dimensionality reduction algorithm, LLE can effectively identify the low dimensional manifold structure that underlies the measured dataset [22]. The LLE algorithm has three basic steps:

Step 1. Compute pairwise distances and find the neighbors of each data point *X_i_*. We defined *X_j_* as one of the neighbors of *X _i_* based on the k-NN algorithm.

Step 2. Compute the weights *W_ij_* that best reconstruct each data point *X_i_* from its neighbors, minimizing the cost by constrained linear fits. We measured reconstruction errors using the cost function
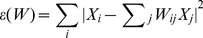
(1)which summed the squared distances between all the data points and their reconstructions. Additionally, the rows of the weight matrix sum to one:



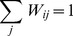
(2)Step 3. Compute the output vectors *Y_i_* best reconstructed by weight *W_ij_*, minimizing the embedding cost function by its lowest d nonzero eigenvectors.
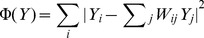
(3)


#### 4.3 Support vector classification

A Support Vector Machine (SVM) classifier aims to find a hyperplane that maximizes the margin between positive and negative samples while simultaneously minimizing misclassification errors in the training set. Decision boundaries can be made non-linear using the so-called “kernel trick” [23]. This method involves mapping the data points onto a higher-dimensional vector space. In this study, we used a radial basis kernel function:
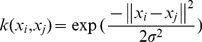
(4)


#### 4.4 Evaluating the performance of the classifiers

The performance of a classifier can be quantified using the generalization rate (GR), sensitivity (SS) and specificity (SC). SS represents the proportion of ASPD correctly predicted ASPD cases, while SC represents the proportion of correctly predicted control cases. The overall proportion of samples correctly predicted is evaluated using the GR. Due to our limited number of samples, we used a leave-one-out cross-validation strategy to estimate the generalization ability of our classifier [24], and all the parameters of the proposed method were optimized based on LOOCV.

To assess the statistical significance of the LOOCV results, we used permutation tests [10], [11], [25], [26]. For permutation testing, the classification labels of the training data were randomly permuted 10,000 times. Cross-validation was then performed on every permuted training set. GR0 was defined as the generalization rate obtained by the classifier trained on the real class labels. When GR0 exceeded the 95% (P<0.05) confidence interval of the classifier trained on randomly re-labeled class labels, it was assumed that the classifier had reliably learned the relationship between the data and the labels. For any value of the estimated GR0, the P-value represented the probability of observing a classification prediction rate of no less than GR0.

## Results

### 1. Classification Results

**Figure 2:**
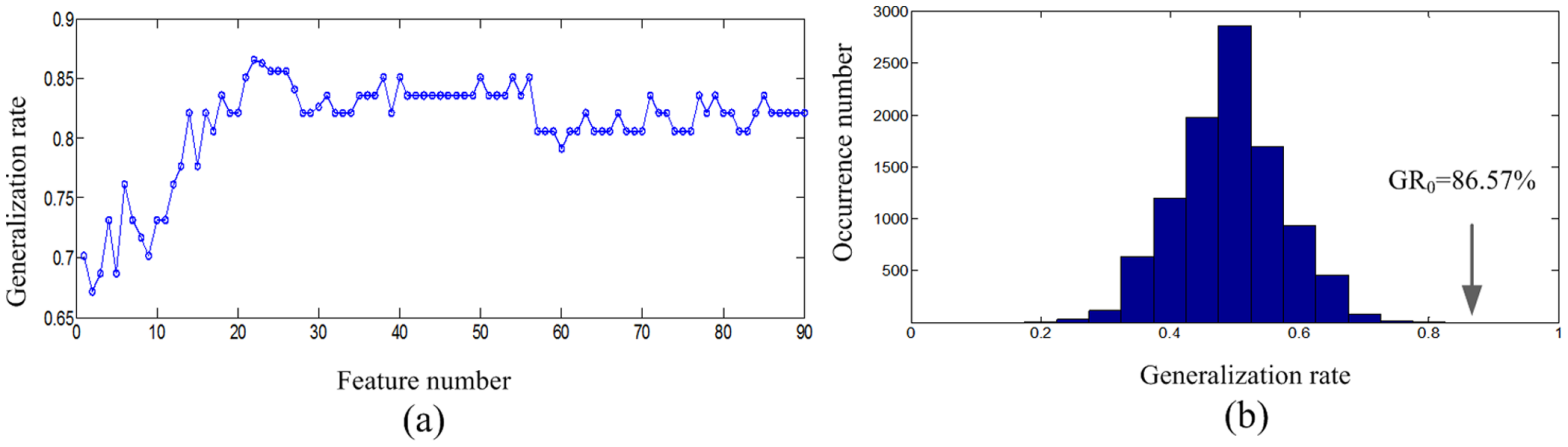
Performance evaluation of the LDA+SVM classifier. (a) The curve of the generalization rate to the number of features. (b) Permutation distribution of the estimate (repetition times: 10,000). GR0 is the generation rate obtained by the classifier trained on the real class labels. With the generalization rate statistic, this figure reveals that the classifier learned the relationship between the data and the labels with a probability of being wrong of <0.0001.

To estimate the effect of the selected parameters on performance of the classifier, we repeated the cross-validation calculation using different parameters [1], [6], [10], [27]. The classifier's best performance (GR: 86.57%, SS: 77.14%, SC: 96.88%) was found at the 22 most discriminating functional connections in the current work (C = 2, neighborhood size: 8, dimensionality: 10) (Figure 2a). Permutation tests revealed that the proposed classifier learned the relationship between the data and the labels with a risk of being wrong of lower than 0.0001 (P<0.0001, Figure 2b).

### 2. Altered Resting-state Functional Connectivity and Networks in ASPD

**Figure 3:**
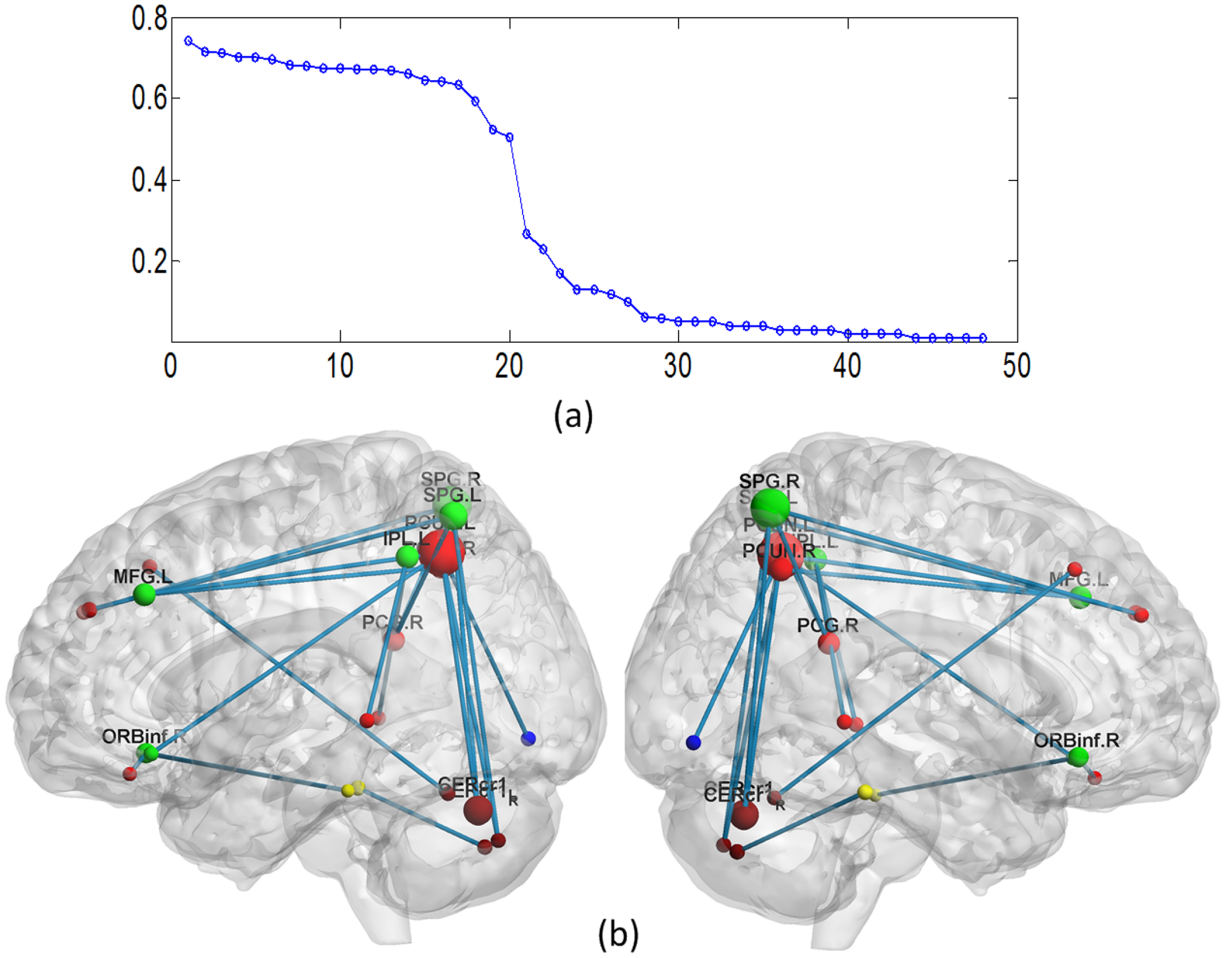
Altered resting-state functional connectivity in ASPD. (a) τ value distribution of all 48 features represented in the LOOCV. The horizontal axis represents each functional connection and the vertical axis represents the weighted Kendall tau correlation coefficient. (b) Region weights and the distribution of the 20 high discriminative power functional connections. Regions are color-coded by category, red sphere represented default mode network, green sphere represented attentional network, brown sphere represented cerebellum.

Because the performance of the classifier was tested using a LOOCV strategy, the functional connectivity feature set selected in each loop was slightly different. Altogether, 48 features were represented during LOOCV when using the 22 most discriminating functional connections to classify ASPD and control subjects. The discriminative power of each feature was computed by multiplying the mean Kendall tau correlation coefficient by the occurrence rate across all iterations of the cross-validation, and 20 features that appeared in no fewer than 51 iterations were found to have high discriminative power (Figure 3). These highly discriminating functional connections represented abnormal resting-state functional connectivity patterns in ASPD. These functional connectivity features with high discriminative power all had positive Kendall tau correlation coefficients, indicating that they decreased in ASPD compared to controls (Table 2). In our study (+1 for controls and −1 for ASPD), a positive correlation coefficient 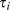 represents a decrease in the *i*th functional connectivity in the ASPD group compared to the control group, and a negative 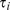
indicates an increase in the *i*th functional connectivity in the ASPD group.

**Table 2:** Altered resting-state functional connectivity and networks in individuals with antisocial personality disorder.

Uncoupled connections	τ value	Uncoupled connections	τ value
**Between default mode and attention**		**Between cerebellar and default mode**	
Frontal Sup Medial (L)/Parietal Sup (L)	0.7411	Precuneus (R)/Cerebelum Crus1 (R)	0.7018
Temporal Mid (L)/Parietal Inf (L)	0.6821	Precuneus (L)/Cerebelum Crus1 (L)	0.6946
Temporal Mid (R)/Parietal Inf (L)	0.6804	Precuneus (L)/Cerebelum Crus2 (L)	0.6696
Frontal Sup Medial (R)/Parietal Sup (L)	0.6750	Frontal Sup (R)/Cerebelum 6 (R)	0.6445
Precuneus (R)/Frontal Mid (L)	0.6598		
Precuneus (R)/Frontal Inf Orb (L)	0.6714	**Between cerebellar and attention**	
Precuneus (R)Frontal Mid (L)	0.6732	Parietal Sup (R)/Cerebelum Crus1 (R)	0.7161
Cingulum Post (R)/Parietal Sup (L)	0.6429	Parietal Sup (L)/Cerebelum Crus1 (L)	0.7125
Cingulum Post (R)/Parietal Sup (R)	0.6330	Parietal Sup (R)/Cerebelum Crus1 (L)	0.5038
Rectus (L)/Frontal Inf Orb (R)	0.5924		
		**Other uncoupled connections**	
		Temporal Inf (R)/Cerebelum Crus2 (R)	0.7018
		Occipital Inf (R)/Precuneus (L)	0.6714
		Frontal Inf Orb (R)/Temporal Inf (L)	0.5227

**Figure 4:**
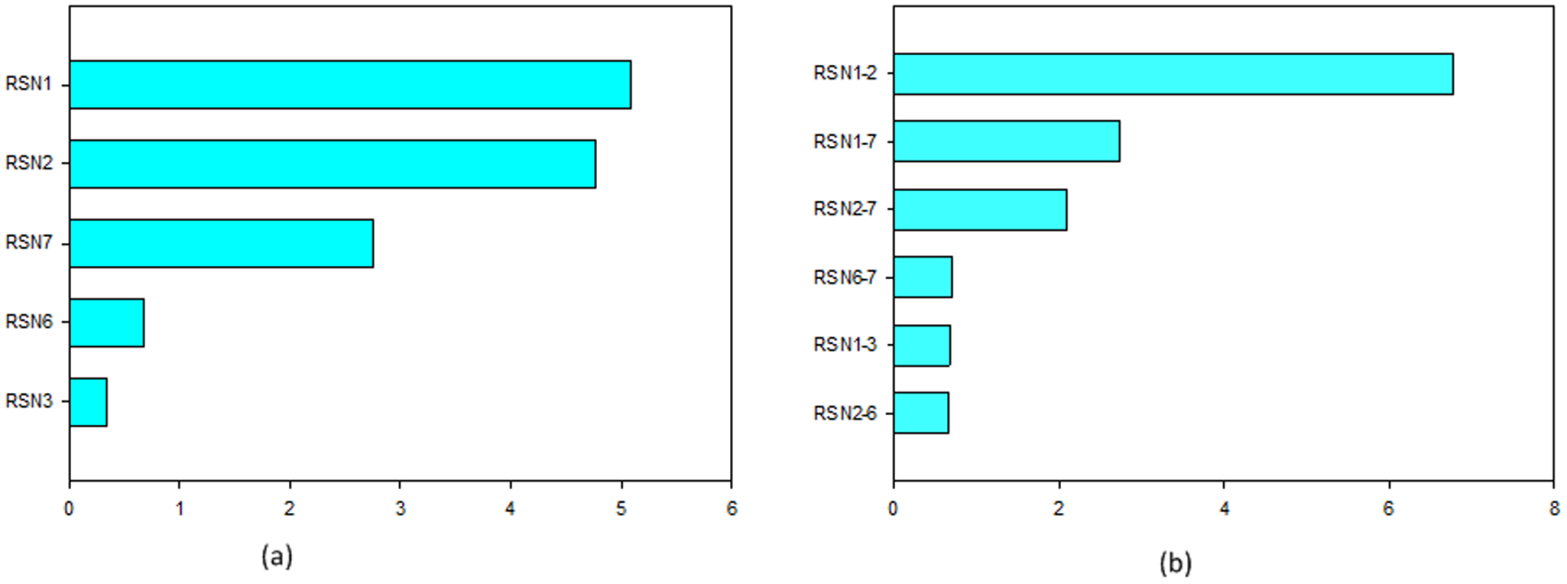
Brain networks weights. (a): Summarized weights for each of the seven communities. (b):The sums of the functional connection weights between the networks. RSN1: default mode network, RSN2: attention network, RSN3, visual recognition network, RSN4: auditory network, RSN5: sensory-motor areas, RSN6: subcortical network, RSN7: cerebellum network.

The 20 functional connectivities with high discriminative power connected 23 brain regions. According to a canonical template of resting-state networks [2], we partitioned the 23 brain regions into seven networks: default mode, attention, visual recognition, auditory, sensory-motor, subcortical and cerebellar. The 20 functional connections were all located between two networks; therefore, half of the feature weight was assigned to each network. Separately summing the feature weights for each network (Figure 4a) revealed that the default mode network and the attention network had much greater sum totals of feature weights, meaning that they had the best relative discriminative powers. The cerebellar network also made a sizeable contribution to discriminating ASPD from control subjects. Moreover, uncoupling of the abnormal functional connections primarily occurred between the default mode network and the attention network (Figure 4b). The cerebellar network also had many uncoupling functional connections with the default mode network and the attention network.

In addition, using two-sample t-tests, we observed significant differences (P<0.05) in 2285 functional connections between ASPD individuals and controls. Some of the connections of them increased and some decreased. When corrected using FDR, 1254 functional connectivities remained that showed significant differences between the two groups, including the 20 abnormal connectivities described in the current paper (P<<0.001). Obviously, the proposed MVPA method reliably found the connectivities with high discriminative power.

### 3. Brain Regions with High Discriminative Power

Region weights were computed by equally dividing the weight of each functional connection between its two constituent regions and then summing all the weights for each of the 23 brain regions (Table 3). For visual representation, the diameter of a sphere, representative of a region, was scaled by the corresponding region weight (Figure 3). The regions with the greatest relative discriminative powers were the precuneus, the superior parietal gyrus and the cerebellum (Crus 1, Crus 2).

**Table 3:** Brain regions with high discriminative power.

Brain region	τ weight	Brain region	τ weight
Precuneus (L)	1.6834	Cerebellum Crus2 (R)	0.3509
Parietal Sup (L)	1.3876	Temporal Mid (L)	0.3411
Cerebellum Crus1 (L)	0.9554	Temporal Mid (R)	0.3402
Parietal Sup (R)	0.9264	Frontal Sup Medial (R)	0.3375
Cerebellum Crus1 (R)	0.7090	Occipital Inf(R)	0.3357
Precuneus (R)	0.6875	Frontal Inf Orb (L)	0.3357
Parietal Inf (L)	0.6813	Cerebellum Crus2 (L)	0.3348
Frontal Mid (L)	0.6665	Frontal Sup Doral(R)	0.3222
Cingulum Post (R)	0.6380	Cerebellum 6 (R)	0.3222
Frontal Inf Orb (R)	0.5576	Rectus (L)	0.2962
Frontal Sup Medial (L)	0.3706	Temporal Inf (L)	0.2614
Temporal Inf (R)	0.3509		

In addition, to investigate whether there were structural abnormalities in subjects with ASPD and whether the functional abnormalities were related to structural abnormalities, a voxel-based morphometry analysis was performed on the T1-weight images. Compared to the controls, the experimental subjects with ASPD had significantly higher gray matter volumes in the parietal lobule, and white matter volumes in the precuneus. See Text S3, Figure S1, Table S1 for structural analysis. Obviously, there are overlapping regions (mainly in the parietal lobule) between the functional and structural brain abnormalities in ASPD.

### 4. Performance Comparison with other Multivariate Pattern Recognition Methods

To better understand the performance of the LLE+SVM classifier, we applied other multivariate pattern recognition methods used in recent studies to identify ASPD individual. We performed 8 additional multivariate pattern classifiers by combining sophisticated dimensionality reduction methods with machine-learning algorithms. In the dimensionality reduction step, we used two approaches, i.e. locally linear embedding (LLE) and principal component analysis (PCA), to reveal the spatiotemporal patterns associated with ASPD. For machine learning, we adopted three sophisticated algorithms, i.e. C-means clustering, SVM with a radial basis kernel function and LDA classification.

To estimate the effects of the selected parameters on the performance of a classifier, we repeated the cross-validation calculation using different parameters [28]. When the optimal parameters of each classifier were selected, optimal performance results of SS, SC and GR were obtained (Table 4). The best result, 86.57% accuracy, was obtained using the LLE+SVM method (77.14% for ASPD and 96.88% for healthy controls).

**Table 4:** Comparison of the classification performance of different multivariate pattern classifiers.

Classifier	Feather	Performance result		
	number	SS(%)	SC(%)	GR(%)
LLE+SVM	22	77.14	96.88	86.57
PCA+SVM	24	71.43	62.5	67.16
SVM	22	80	81.25	80.6
LLE+LDA	21	71.43	93.75	82.09
PCA+LDA	21	80	53.13	67.16
LDA	9	88.57	56.25	73.13
LLE+C-means	61	74.29	84.38	79.1
PCA+C-means	83	15.63	100	59.7
C-means	20	78.13	85.71	82.09

LLE, locally linear embedding; LDA, linear discriminant analysis; PCA, principal component analysis; SVM, Support Vector Machine; GR, generalization rate; SS, sensitivity; SC specificity.

## Discussion

### 1. Altered Resting-state Functional Connectivity in ASPD Individuals

The main contribution of this study was the use of resting–state functional connectivities as classification features to discriminate ASPD individuals from normal controls. The results not only give insight into the pathological mechanisms of this complex mental disorder from a resting-state functional integration viewpoint, but also provided evidence of functional disconnection. ASPD was associated with the altered functional connections with high discriminative power, which were mostly located between the default mode network and attention network, or between those networks and the cerebellar network. In particular, the precuneus, superior parietal gyrus and cerebellum exhibited high discriminative power in our classification. A voxel-based morphometry analysis of the T1-weight images revealed that gray matter volumes in the parietal lobule and white matter volumes in the precuneus were abnormal in ASPD subjects compared to controls. Clearly, there are overlapping regions between functional and structural brain abnormalities in ASPD. The functional abnormalities may be related to the structural abnormalities.

#### 1.1 Default mode network

In our study, the regions that were correlated with the uncoupled connections in the default mode network included the precuneus, posterior cingulate cortex, superior frontal gyrus, middle temporal gyrus, and rectus gyrus. The default mode network is hypothesized to perform functions, such as emotional regulation, related to planning for the future using past experiences, and self-inspection [29]–[32]. Decreased functioning of the default mode network may manifest as difficulties in adaptively regulating emotions, future planning or self-inspection. Individuals with ASPD demonstrated decreased functional connectivity between regions of the default mode and attention networks, and this could be interpreted as inefficient transmission between the default mode network, which detects conflict, and the attention network, which implements increased cognitive control to resolve conflict in future trials. There was also decreased functional connectivity between regions of the default mode network and regions of the cerebellar network.

In the default mode network, the precuneus was found to have the highest discriminative power, followed by the posterior cingulated gyrus, the superior frontal gyrus and the middle temporal gyrus. The functional abnormalities in the precuneus were consistent with our structural finding that the precuneus was abnormal in ASPD in T1-weighted images. It has been suggested that together with the posterior cingulate, the precuneus is “pivotal for conscious information processing” [33], [34] and is the “core node” or “hub” of the default mode network [33], [35]. The precuneus is involved in the processes of self-consciousness, such as reflective self-awareness [36], [37]. Previous neuroimaging studies have concluded that the precuneus/posterior cingulate is involved in a range of cognitive tasks that touches upon various aspects of self-processing [38], [39], and is essential for conscious awareness [40]. Goldberg et al. have found evidence that the superior frontal gyrus is involved in self-awareness, in coordination with the sensory system [41], [42]. The superior frontal gyrus is the primary brain locus that implements the set of functional operations subtended in task-set reconfiguration operations [43], [44]. Jastorff et al. found that the main cognitive component underlying middle temporal gyrus activation in their study was the evaluation of action rationality [45]. The temporal lobes take part in sensory, affective, and higher cognitive processing [46].

Taken together, these converging lines of evidence suggest that if those areas of the default mode network do not function properly, a person may act impulsively and inappropriately, producing antisocial emotion. The associated inability to act in a “civilized” manner often results in criminality.

#### 1.2 Attention network

The regions that were correlated with uncoupled connections in ASPD in the attention network were the superior and inferior parietal cortices, the inferior frontal gyrus (opercula), and the middle frontal gyrus. The attention network is involved in directing attention to new stimuli [47]–[50], and may be responsible for implementing cognitive control [47], [49], [51]–[53]. Recently, researchers proposed that the attention network is also related to self-regulation [54]–[57]. Decreased functioning between the attention network and the fault mode network or cerebellar network may result in deficits of transmission in the implementation of cognitive control and self-regulation. High levels of effortful control and the ability to resolve conflict are related to fewer antisocial behaviors in adolescents [58].

In the attention network, the superior parietal gyrus was found to have the highest discriminative power, followed by the inferior parietal gyrus, and the inferior and middle frontal gyri. We also found the structural abnormalities in the inferior parietal lobule in ASPD in T1-weighted images. The superior parietal cortex is critically important for the manipulation of information in working memory [59] and for rule-based visual-motor transformations [60]. The superior parietal lobe is also critical for sensorimotor integration, via maintenance of an internal representation of the body's state [61]. The inferior parietal lobule has been involved in the perception of emotions in facial stimuli [62] and plays a key role in various cognitive functions, including attention, language, and action processing [63]. The right inferior frontal gyrus is critically important for response inhibition [64]–[67]. Disruption of activity in this area using transcranial magnetic stimulation or direct current stimulation leads to changes in risk attitudes, as behaviorally demonstrated by choices of risky outcomes [68], [69]. The left inferior frontal gyrus is extremely important for language production and verb comprehension. The origin of the human motor readiness fields is linked to the left middle frontal gyrus [70]. The functional abnormalities in the inferior parietal lobule were consistent with our structural finding that the precuneus was abnormal in ASPD in T1-weighted images. The left middle frontal gyrus was also involved in phonological and semantic tasks [71].

The functions of the above regions are important for the selection and control of socially relevant behavior. When these functions are impaired, the other cognitive systems may be affected. Injury to these lobes may cause violent and aggressive behavior or the development of cold-bloodedness, which are major characteristics of ASPD.

#### 1.3 Cerebellum

In the current study, altered connections were also observed between the cerebellum and the regions in the default mode network and attention network. The first function of the cerebellum is to organize complex information received by the brain. It coordinates basic memory and learning processes and is involved in emotional and cognitive processes, such as attention, fear regulation and pleasure responses [72]–[76]. Within the cerebellum, crus1 and crus2 were found to have the highest discriminative power. Crus1 and crus2 were involved in higher-level tasks [77] and contributed to complex cognitive operations [78]–[81]. Executive tasks activated regions of crus1 that are proposed to be involved in prefrontal-cerebellar loops [77] and working memory [82]. Cerebro-cerebellar circuits may underlie the involvement of the cerebellum in executive functions [83]. We speculate that the aberrant cerebellar connectivity with the default mode and attention networks may be partially involved in the emotional and cognitive symptoms of ASPD.

In this study, all of the examined brain regions are related to symptoms of depersonalization. The functional and structural deficits may underlie the low arousal, high impulsivity, lack of conscience, cold-bloodedness and decision-making deficits of seen in ASPD. ASPD may be characterized by uncoupling of functional connectivity patterns at the network level. Many behavioral changes in ASPD may be associated with individual networks, and ASPD may arise as an interaction between these behaviors. Our observations extend the current understanding of the neuroanatomical features of ASPD.

### 2. Reliability of the Proposed Classifier

In the present study, a LLE+SVM classifier was designed to classify the resting-state functional connectivity of ASPD and exhibited a satisfactory correct classification rate. We used the Kendall tau rank correlation coefficient [1], [21], which can quantitatively measure a feature’s relevance to classification, to produces a subset of the original features. Then, an LLE-based dimensionality reduction was performed to find a low-dimensional representation of the abnormal resting-state functional connectivity patterns in ASPD patients in contrast to controls. A SVM classifier was used to finding a hyperplane, and LOOCV was adopted to estimate performance and optimize the parameters. The LLE+SVM classifier significantly outperformed the other tested classifiers (Table 4). LLE dimensionality reduction significantly outperformed the PCA linear dimensionality reduction. This might be ascribed to the essentially nonlinear neural dynamics underlying resting-state brain activities. The similar conclusion was also obtained in the discrimination of schizophrenia [6]. LLE transforms data space and results in an intrinsically low-dimensional structure. When the GR of the LLE+SVM classifier and the other four classifiers with GRs greater than 80% (Table 4) arrived the peak point, the number of features ranged from 20–23. This clearly showed the abnormal information abstracted by the proposed classifier was consistent with that abstracted by the other classifiers. Importantly, even when the number of features was not optimized or in a wide range (20–300), the poorest result of the proposed classifier was approximately 80% (Figure 2). This showed that the proposed classifier had high stability under noisy conditions. The results of permutation test also revealed that the proposed classifier reliably learned the relationship between the data and the labels.

This study not only demonstrated high classification accuracy of the LLE+SVM classifier, but also elucidated the pathological mechanisms of ASPD from a resting-state functional integration viewpoint. In future work, we will test the method on a larger independent dataset to confirm our findings.

## Supporting Information

Figure S1 **Results of a Voxel-based Morphometry Analysis.** (A) Statistic parametric map in three orthogonal projections shows voxels where a higher regional gray-matter density emerged in ASPD vs. control images. The voxel of maximal gray matter density was at [x, y, z] = (41, −40, 48). (B) Statistic parametric map in three orthogonal projections shows voxels where a higher regional white-matter density emerged in ASPD vs. control images. The voxel of maximal gray matter density was at [x, y, z] = (24, −58, 40). ASPD: antisocial personality disorder.
(TIF)

Table S1 **The Abnormal Brain Regions in ASPD vs. Controls by a Voxel-based Morphometry Analysis.** These results were produced with an uncorrected voxel level height threshold of P≤0.001 and a cluster threshold >70.
(DOC)

Text S1 **The Details of the Informed Consent Procedures.**
(DOC)

Text S2 **Kendall Tau Rank Correlation Coefficient.**
(DOC)

Text S3 **A Voxel-based Morphometry Analysis and Results.**
(DOC)

## References

[1] Zeng LL, Shen H, Liu L, Wang L, Li B, et al (2012) Identifying major depression using whole-brain functional connectivity: a multivariate pattern analysis. Brain 135: 1498–1507. 22418737 10.1093/brain/aws059

[2] Tao H, Guo S, Ge T, Kendrick KM, Xue Z, et al. (2011) Depression uncouples brain hate circuit. Mol Psychiatry. 10.1038/mp.2011.127 PMC3526729 21968929

[3] Sheline YI, Price JL, Yan Z, Mintun MA (2010) Resting-state functional MRI in depression unmasks increased connectivity between networks via the dorsal nexus. Proc Natl Acad Sci U S A 107: 11020–11025. 20534464 10.1073/pnas.1000446107 PMC2890754

[4] Greicius MD, Srivastava G, Reiss AL, Menon V (2004) Default-mode network activity distinguishes Alzheimer's disease from healthy aging: evidence from functional MRI. Proc Natl Acad Sci U S A 101: 4637–4642. 15070770 10.1073/pnas.0308627101 PMC384799

[5] Zhou J, Greicius MD, Gennatas ED, Growdon ME, Jang JY, et al (2010) Divergent network connectivity changes in behavioural variant frontotemporal dementia and Alzheimer's disease. Brain 133: 1352–1367. 20410145 10.1093/brain/awq075 PMC2912696

[6] Shen H, Wang L, Liu Y, Hu D (2010) Discriminative analysis of resting-state functional connectivity patterns of schizophrenia using low dimensional embedding of fMRI. NeuroImage 49: 3110–3121. 19931396 10.1016/j.neuroimage.2009.11.011

[7] Whitfield-Gabrieli S, Thermenos HW, Milanovic S, Tsuang MT, Faraone SV, et al (2009) Hyperactivity and hyperconnectivity of the default network in schizophrenia and in first-degree relatives of persons with schizophrenia. Proc Natl Acad Sci U S A 106: 1279–1284. 19164577 10.1073/pnas.0809141106 PMC2633557

[8] Liu Y, Liang M, Zhou Y, He Y, Hao Y, et al (2008) Disrupted small-world networks in schizophrenia. Brain 131: 945–961. 18299296 10.1093/brain/awn018

[9] Wang Q, Su TP, Zhou Y, Chou KH, Chen IY, et al (2012) Anatomical insights into disrupted small-world networks in schizophrenia. Neuroimage 59: 1085–1093. 21963918 10.1016/j.neuroimage.2011.09.035

[10] Dosenbach NU, Nardos B, Cohen AL, Fair DA, Power JD, et al (2010) Prediction of individual brain maturity using fMRI. Science 329: 1358–1361. 20829489 10.1126/science.1194144 PMC3135376

[11] Golland P, Fischl B (2003) Permutation tests for classification: towards statistical significance in image-based studies. Inf Process Med Imaging 18: 330–341. 15344469 10.1007/978-3-540-45087-0_28

[12] Widiger TA, Costa PT Jr (1994) Personality and personality disorders. J Abnorm Psychol 103: 78–91. 8040485 10.1037//0021-843x.103.1.78

[13] Samuel DB, Widiger TW (2010) Comparing personality disorder models: cross-method assessment of the FFM and DSM-IV-TR. J Pers Disord 24: 721–745. 21158596 10.1521/pedi.2010.24.6.721 PMC3007669

[14] Association AP (1994) Diagnostic and Statistical Manual of Mental Disorders (4th edn)(DSM-IV). Washington,DC: APA.

[15] Tzourio-Mazoyer N, Landeau B, Papathanassiou D, Crivello F, Etard O, et al (2002) Automated anatomical labeling of activations in SPM using a macroscopic anatomical parcellation of the MNI MRI single-subject brain. NeuroImage 15: 273–289. 11771995 10.1006/nimg.2001.0978

[16] Shen H, Wang L, Liu Y, Hu D (2010) Discriminative analysis of resting-state functional connectivity patterns of schizophrenia using low dimensional embedding of fMRI. NeuroImage 49: 3110–3121. 19931396 10.1016/j.neuroimage.2009.11.011

[17] Fox MD, Zhang D, Snyder AZ, Raichle ME (2009) The global signal and observed anticorrelated resting state brain networks. J Neurophysiol 101: 3270–3283. 19339462 10.1152/jn.90777.2008 PMC2694109

[18] Fair DA, Cohen AL, Dosenbach NU, Church JA, Miezin FM, et al (2008) The maturing architecture of the brain's default network. Proc Natl Acad Sci U S A 105: 4028–4032. 18322013 10.1073/pnas.0800376105 PMC2268790

[19] Biswal BB, Mennes M, Zuo XN, Gohel S, Kelly C, et al (2010) Toward discovery science of human brain function. Proc Natl Acad Sci U S A 107: 4734–4739. 20176931 10.1073/pnas.0911855107 PMC2842060

[20] Guyon I, Elisseeff A (2003) An introduction to variable and feature selection. The Journal of Machine Learning Research 3: 1157–1182.

[21] Govindarajulu Z (1992) Rank Correlation Methods. Technometrics 34: 108–108.

[22] Roweis ST, Saul LK (2000) Nonlinear dimensionality reduction by locally linear embedding. Science 290: 2323–2326. 11125150 10.1126/science.290.5500.2323

[23] Ben-Hur A, Ong CS, Sonnenburg S, Schölkopf B, Rätsch G (2008) Support vector machines and kernels for computational biology. PLoS Comput Biol. 4, e1000173. 10.1371/journal.pcbi.1000173 PMC2547983 18974822

[24] Zhu CZ, Zang YF, Cao QJ, Yan CG, He Y, et al (2008) Fisher discriminative analysis of resting-state brain function for attention-deficit/hyperactivity disorder. Neuroimage 40: 110–120. 18191584 10.1016/j.neuroimage.2007.11.029

[25] Meriaux S, Roche A, Dehaene-Lambertz G, Thirion B, Poline JB (2006) Combined permutation test and mixed-effect model for group average analysis in fMRI. Hum Brain Mapp 27: 402–410. 16596617 10.1002/hbm.20251 PMC6871503

[26] Nichols TE, Holmes AP (2002) Nonparametric permutation tests for functional neuroimaging: a primer with examples. Hum Brain Mapp 15: 1–25. 11747097 10.1002/hbm.1058 PMC6871862

[27] Tang Y, Wang L, Cao F, Tan L (2012) Identify schizophrenia using resting-state functional connectivity: an exploratory research and analysis. Biomed Eng Online 11: 50. 22898249 10.1186/1475-925X-11-50 PMC3462724

[28] (!!! INVALID CITATION !!!).

[29] Raichle ME, MacLeod AM, Snyder AZ, Powers WJ, Gusnard DA, et al (2001) A default mode of brain function. Proceedings of the National Academy of Sciences 98: 676–682. 10.1073/pnas.98.2.676 PMC14647 11209064

[30] Sylvester CM, Corbetta M, Raichle ME, Rodebaugh TL, Schlaggar BL, et al (2012) Functional network dysfunction in anxiety and anxiety disorders. Trends Neurosci 35: 527–535. 22658924 10.1016/j.tins.2012.04.012 PMC3432139

[31] Delgado MR, Nearing KI, LeDoux JE, Phelps EA (2008) Neural circuitry underlying the regulation of conditioned fear and its relation to extinction. Neuron 59: 829–838. 18786365 10.1016/j.neuron.2008.06.029 PMC3061554

[32] Buckner RL, Andrews-Hanna JR, Schacter DL (2008) The brain's default network. Annals of the New York Academy of Sciences 1124: 1–38. 18400922 10.1196/annals.1440.011

[33] Cavanna AE (2007) The precuneus and consciousness. CNS Spectr 12: 545–552. 17603406 10.1017/s1092852900021295

[34] Vogt BA, Laureys S (2005) Posterior cingulate, precuneal and retrosplenial cortices: cytology and components of the neural network correlates of consciousness. Prog Brain Res 150: 205–217. 16186025 10.1016/S0079-6123(05)50015-3 PMC2679949

[35] Buckner RL, Andrews-Hanna JR, Schacter DL (2008) The brain's default network: anatomy, function, and relevance to disease. Ann N Y Acad Sci 1124: 1–38. 18400922 10.1196/annals.1440.011

[36] Kjaer TW, Nowak M, Lou HC (2002) Reflective self-awareness and conscious states: PET evidence for a common midline parietofrontal core. Neuroimage 17: 1080–1086. 12377180

[37] Lou HC, Luber B, Crupain M, Keenan JP, Nowak M, et al (2004) Parietal cortex and representation of the mental Self. Proc Natl Acad Sci U S A 101: 6827–6832. 15096584 10.1073/pnas.0400049101 PMC404216

[38] Cavanna AE, Trimble MR (2006) The precuneus: a review of its functional anatomy and behavioural correlates. Brain 129: 564–583. 16399806 10.1093/brain/awl004

[39] Buckner RL, Carroll DC (2007) Self-projection and the brain. Trends Cogn Sci 11: 49–57. 17188554 10.1016/j.tics.2006.11.004

[40] Vogt BA, Laureys S (2005) Posterior cingulate, precuneal and retrosplenial cortices: cytology and components of the neural network correlates of consciousness. 150: 205–217. 10.1016/S0079-6123(05)50015-3 PMC2679949 16186025

[41] Vince G (2006) Watching the brain ‘switch off’ self-awareness. NewScientist. Available: http://www.newscientist.com/article/dn9019.

[42] Goldberg II Goldberg, II, Harel M, Malach R (2006) When the brain loses its self: prefrontal inactivation during sensorimotor processing. Neuron 50: 329–339. 16630842 10.1016/j.neuron.2006.03.015

[43] Crone EA, Wendelken C, Donohue SE, Bunge SA (2006) Neural evidence for dissociable components of task-switching. Cereb Cortex 16: 475–486. 16000652 10.1093/cercor/bhi127

[44] Cutini S, Scatturin P, Menon E, Bisiacchi PS, Gamberini L, et al (2008) Selective activation of the superior frontal gyrus in task-switching: an event-related fNIRS study. NeuroImage 42: 945–955. 18586525 10.1016/j.neuroimage.2008.05.013

[45] Jastorff J, Clavagnier S, Gergely G, Orban GA (2011) Neural mechanisms of understanding rational actions: middle temporal gyrus activation by contextual violation. Cereb Cortex 21: 318–329. 20513657 10.1093/cercor/bhq098

[46] Kolb B, Whishaw I (1990) Fundamentals of Human Neuropsychology. WH Freeman and Co, New York.

[47] Petersen SE, Posner MI (2012) The attention system of the human brain: 20 years after. Annu Rev Neurosci 35: 73–89. 22524787 10.1146/annurev-neuro-062111-150525 PMC3413263

[48] Lindner A, Iyer A, Kagan I, Andersen RA (2010) Human posterior parietal cortex plans where to reach and what to avoid. J Neurosci 30: 11715–11725. 20810892 10.1523/JNEUROSCI.2849-09.2010 PMC2956133

[49] Posner MI, Petersen SE (1989) The attention system of the human brain. DTIC Document.

[50] Corbetta M, Shulman GL (2002) Control of goal-directed and stimulus-driven attention in the brain. Nature Reviews Neuroscience 3: 215–229. 10.1038/nrn755 11994752

[51] Carter CS, Krug MK (2011) Dynamic Cognitive Control and Frontal–Cingulate Interactions. Cognitive Neuroscience of Attention: 89.

[52] Power JD, Cohen AL, Nelson SM, Wig GS, Barnes KA, et al (2011) Functional network organization of the human brain. Neuron 72: 665–678. 22099467 10.1016/j.neuron.2011.09.006 PMC3222858

[53] Bush G, Luu P, Posner MI (2000) Cognitive and emotional influences in anterior cingulate cortex. Trends in cognitive sciences 4: 215–222. 10827444 10.1016/s1364-6613(00)01483-2

[54] Rothbart MK, Sheese BE, Rueda MR, Posner MI (2011) Developing mechanisms of self-regulation in early life. Emotion review 3: 207–213. 21892360 10.1177/1754073910387943 PMC3164871

[55] Beauregard M, Levesque J, Bourgouin P (2001) Neural correlates of conscious self-regulation of emotion. The Journal of Neuroscience. 10.1523/JNEUROSCI.21-18-j0001.2001 PMC6763007 11549754

[56] Ochsner KN, Bunge SA, Gross JJ, Gabrieli JDE (2002) Rethinking feelings: An fMRI study of the cognitive regulation of emotion. Journal of cognitive neuroscience 14: 1215–1229. 12495527 10.1162/089892902760807212

[57] Posner MI, Rothbart MK (2007) Research on attention networks as a model for the integration of psychological science. Annu Rev Psychol 58: 1–23. 17029565 10.1146/annurev.psych.58.110405.085516

[58] Rothbart MK (2011) Becoming who we are. Guilford, New York.

[59] Koenigs M, Barbey AK, Postle BR, Grafman J (2009) Superior parietal cortex is critical for the manipulation of information in working memory. J Neurosci 29: 14980–14986. 19940193 10.1523/JNEUROSCI.3706-09.2009 PMC2799248

[60] Hawkins KM, Sayegh P, Yan X, Crawford JD, Sergio LE (2012) Neural Activity in Superior Parietal Cortex during Rule-based Visual-motor Transformations. J Cogn Neurosci. 10.1162/jocn_a_00318 23092356

[61] Wolpert DM, Goodbody SJ, Husain M (1998) Maintaining internal representations: the role of the human superior parietal lobe. Nat Neurosci 1: 529–533. 10196553 10.1038/2245

[62] Radua J, Phillips ML, Russell T, Lawrence N, Marshall N, et al (2010) Neural response to specific components of fearful faces in healthy and schizophrenic adults. NeuroImage 49: 939–946. 19699306 10.1016/j.neuroimage.2009.08.030

[63] Caspers S, Schleicher A, Bacha-Trams M, Palomero-Gallagher N, Amunts K, et al. (2012) Organization of the Human Inferior Parietal Lobule Based on Receptor Architectonics. Cereb Cortex. 10.1093/cercor/bhs048 PMC3563340 22375016

[64] Forstmann BU, Jahfari S, Scholte HS, Wolfensteller U, van den Wildenberg WP, et al (2008) Function and structure of the right inferior frontal cortex predict individual differences in response inhibition: a model-based approach. J Neurosci 28: 9790–9796. 18815263 10.1523/JNEUROSCI.1465-08.2008 PMC6671204

[65] Aron AR, Robbins TW, Poldrack RA (2004) Inhibition and the right inferior frontal cortex. Trends Cogn Sci 8: 170–177. 15050513 10.1016/j.tics.2004.02.010

[66] Lopez-Caneda E, Cadaveira F, Crego A, Gomez-Suarez A, Corral M, et al (2012) Hyperactivation of right inferior frontal cortex in young binge drinkers during response inhibition: a follow-up study. Addiction 107: 1796–1808. 22487028 10.1111/j.1360-0443.2012.03908.x

[67] Christopoulos GI, Tobler PN, Bossaerts P, Dolan RJ, Schultz W (2009) Neural correlates of value, risk, and risk aversion contributing to decision making under risk. J Neurosci 29: 12574–12583. 19812332 10.1523/JNEUROSCI.2614-09.2009 PMC2794196

[68] Knoch D, Gianotti LR, Pascual-Leone A, Treyer V, Regard M, et al (2006) Disruption of right prefrontal cortex by low-frequency repetitive transcranial magnetic stimulation induces risk-taking behavior. J Neurosci 26: 6469–6472. 16775134 10.1523/JNEUROSCI.0804-06.2006 PMC6674035

[69] Fecteau S, Pascual-Leone A, Zald DH, Liguori P, Theoret H, et al (2007) Activation of prefrontal cortex by transcranial direct current stimulation reduces appetite for risk during ambiguous decision making. J Neurosci 27: 6212–6218. 17553993 10.1523/JNEUROSCI.0314-07.2007 PMC6672163

[70] Pedersen JR, Johannsen P, Bak CK, Kofoed B, Saermark K, et al (1998) Origin of human motor readiness field linked to left middle frontal gyrus by MEG and PET. NeuroImage 8: 214–220. 9740763 10.1006/nimg.1998.0362

[71] Liu CL, Hue CW, Chen CC, Chuang KH, Liang KC, et al (2006) Dissociated roles of the middle frontal gyri in the processing of Chinese characters. Neuroreport 17: 1397–1401. 16932146 10.1097/01.wnr.0000233090.00463.35

[72] Dolan RJ (1998) A cognitive affective role for the cerebellum. Brain 121 (Pt 4): 545–546. 10.1093/brain/121.4.545 9577383

[73] Partridge J, Rayner J, Awan S (2010) The cerebellar cognitive affective syndrome. Br J Hosp Med (Lond) 71: 712–713. 21135772 10.12968/hmed.2010.71.12.712

[74] O'Reilly JX, Beckmann CF, Tomassini V, Ramnani N, Johansen-Berg H (2010) Distinct and overlapping functional zones in the cerebellum defined by resting state functional connectivity. Cereb Cortex 20: 953–965. 19684249 10.1093/cercor/bhp157 PMC2837094

[75] Moulton EA, Elman I, Pendse G, Schmahmann J, Becerra L, et al (2011) Aversion-related circuitry in the cerebellum: responses to noxious heat and unpleasant images. J Neurosci 31: 3795–3804. 21389234 10.1523/JNEUROSCI.6709-10.2011 PMC3063442

[76] Wolf U, Rapoport MJ, Schweizer TA (2009) Evaluating the affective component of the cerebellar cognitive affective syndrome. J Neuropsychiatry Clin Neurosci 21: 245–253. 19776302 10.1176/jnp.2009.21.3.245

[77] Stoodley CJ, Schmahmann JD (2009) Functional topography in the human cerebellum: a meta-analysis of neuroimaging studies. Neuroimage 44: 489–501. 18835452 10.1016/j.neuroimage.2008.08.039

[78] Exner C, Weniger G, Irle E (2004) Cerebellar lesions in the PICA but not SCA territory impair cognition. Neurology 63: 2125–2132. 10.1212/01.wnl.0000146197.44568.cd 15596762

[79] Schmahmann JD, Weilburg JB, Sherman JC (2007) The neuropsychiatry of the cerebellum - insights from the clinic. Cerebellum 6: 254–267. 17786822 10.1080/14734220701490995

[80] Schoch B, Dimitrova A, Gizewski ER, Timmann D (2006) Functional localization in the human cerebellum based on voxelwise statistical analysis: a study of 90 patients. Neuroimage 30: 36–51. 16253526 10.1016/j.neuroimage.2005.09.018

[81] Tavano A, Grasso R, Gagliardi C, Triulzi F, Bresolin N, et al (2007) Disorders of cognitive and affective development in cerebellar malformations. Brain 130: 2646–2660. 17872929 10.1093/brain/awm201

[82] Chen SH, Desmond JE (2005) Cerebrocerebellar networks during articulatory rehearsal and verbal working memory tasks. Neuroimage 24: 332–338. 15627576 10.1016/j.neuroimage.2004.08.032

[83] Bellebaum C, Daum I (2007) Cerebellar involvement in executive control. Cerebellum 6: 184–192. 17786814 10.1080/14734220601169707

