# Correction: Identifying Individuals with Antisocial Personality Disorder Using Resting-State fMRI

**DOI:** 10.1371/journal.pone.0096962

**Published:** 2014-04-29

**Authors:** The PLOS ONE Staff

Yan Tang and Weixiong Jiang should be noted as co-first authors and have contributed equally to this work. Yan Tang and Weixiong Jiang are incorrectly noted as corresponding authors. Wei Wang is the sole Corresponding author for this article and can be reached by email here: wawe01cn@yahoo.com.cn

The publisher apologizes for this error.
