# Supplementary figures and images for: Identifying Individuals with Antisocial Personality Disorder Using Resting-State fMRI
Source: PLoS One. 2013 Apr 12;8(4):e60652. doi: 10.1371/journal.pone.0060652 (PMC3625191; doi:10.1371/journal.pone.0060652)

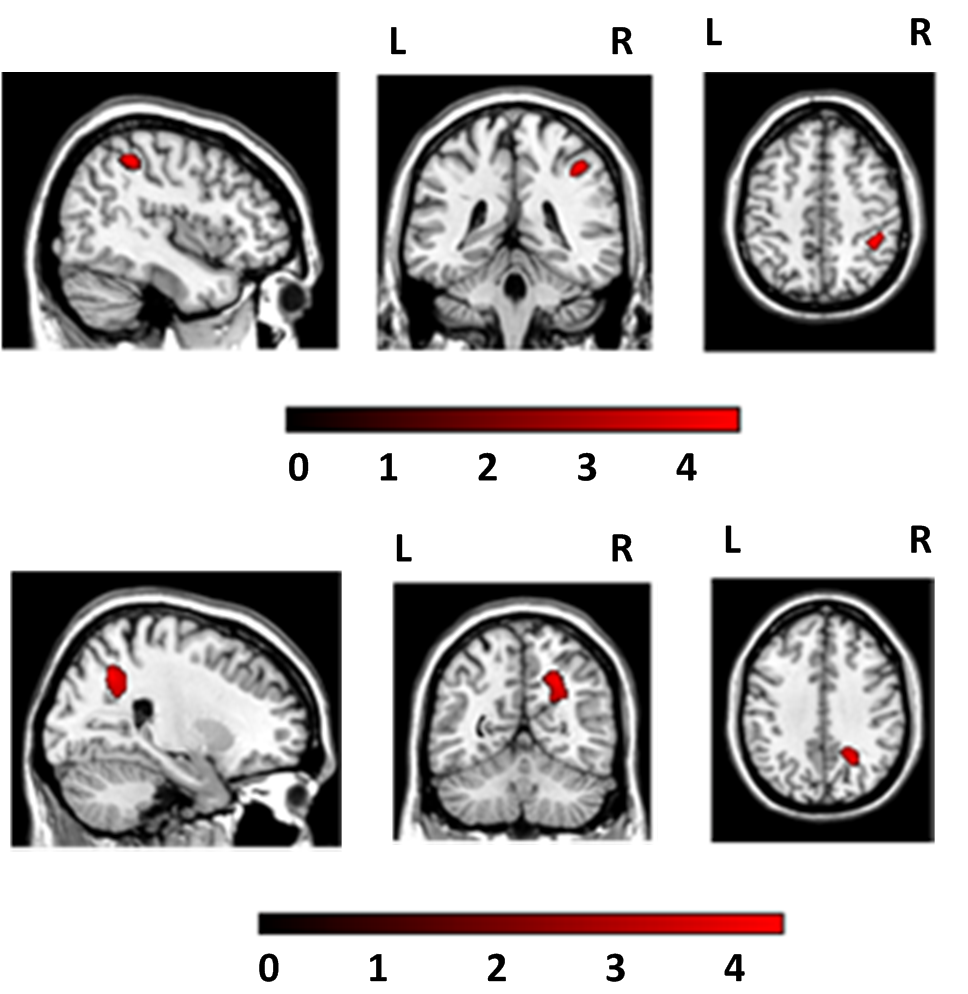

Supplement: Figure S1 — Results of a Voxel-based Morphometry Analysis. (A) Statistic parametric map in three orthogonal projections shows voxels where a higher regional gray-matter density emerged in ASPD vs. control images. The voxel of maximal gray matter density was at [x, y, z] = (41, −40, 48). (B) Statistic parametric map in three orthogonal projections shows voxels where a higher regional white-matter density emerged in ASPD vs. control images. The voxel of maximal gray matter density was at [x, y, z] = (24, −58, 40). ASPD: antisocial personality disorder. (TIF) [file pone.0060652.s001.tif]
