# Supplementary material for: Identifying Individuals with Antisocial Personality Disorder Using Resting-State fMRI
Source: PLoS One. 2013 Apr 12;8(4):e60652. doi: 10.1371/journal.pone.0060652 (PMC3625191; doi:10.1371/journal.pone.0060652)
Supplement: Table S1 — The Abnormal Brain Regions in ASPD vs. Controls by a Voxel-based Morphometry Analysis. These results were produced with an uncorrected voxel level height threshold of P≤0.001 and a cluster threshold >70. (DOC) [file pone.0060652.s002.doc]

**Table S1. The Abnormal Brain Regions in ASPD vs. Controls by a Voxel-based Morphometry Analysis. These results were produced with an uncorrected voxel level height threshold of P≤0.001and a cluster threshold >70.**

| Region | MNI Coordinates^c^ | | | z-value | Cluster | p value |
| --- | --- | --- | --- | --- | --- | --- |
|  | x | y | z |  | size^a^ | (peak-level)^b^ |
| **Gray matter volume（ASPD>controls）** |  |  |  |  |  |  |
| Right , Inferior Parietal Lobule | 41 | -40 | 48 | 4.00 | 216 | 0.000 |
| **White matter volume（ASPD>controls）** |  |  |  |  |  |  |
| Right,Precuneus | 24 | -58 | 40 | 4.29 | 648 | 0.000 |

a Total number of contiguous voxels in each region

b Z-score for the voxel of maximal statistical significance in each region.

c Coordinates of the voxel of maximal statistical significance within each region.
