# Supplementary material for: Identifying Individuals with Antisocial Personality Disorder Using Resting-State fMRI
Source: PLoS One. 2013 Apr 12;8(4):e60652. doi: 10.1371/journal.pone.0060652 (PMC3625191; doi:10.1371/journal.pone.0060652)
Supplement: Text S1 — The Details of the Informed Consent Procedures. (DOC) [file pone.0060652.s003.doc]

**Text S1: The Details of the Informed Consent Procedures**

We first introduced MRI knowledge and other relevant knowledge to every participant, particularly the contraindications for and discomfort of MRI scanning, and all were informed to be free to decide whether to participate or not, they could also stop participating at any phase. We guaranteed that all potential participants who declined to participate or otherwise did not participate were not disadvantaged in any other way by not participating in the study. Then we provided a detailed description of the experimental procedure. Finally the written informed consent was completed, which confirmed that the study was safe, and had no commercial motivation; it also confirmed that participants’ privacy would be protected. Participants were paid a base rate of ¥100 for their participation, plus an additional ¥50 bonus according to their performance.

Written informed consents for the study and publishing these case details were obtained from all the subjects. This study was approved by the Ethical Committee of the Third Xiangya Hospital of Central South University and the School for Youthful Offender of Hunan Province.
