# Supplementary material for: Identifying Individuals with Antisocial Personality Disorder Using Resting-State fMRI
Source: PLoS One. 2013 Apr 12;8(4):e60652. doi: 10.1371/journal.pone.0060652 (PMC3625191; doi:10.1371/journal.pone.0060652)
Supplement: Text S2 — Kendall Tau Rank Correlation Coefficient. (DOC) [file pone.0060652.s004.doc]

**Text S2: Kendall Tau Rank Correlation Coefficient**

Suppose that there are *m* samples in the ASPD group and *n* samples in the control group. Let $x_{ij}$ denotes the functional connectivity feature $i$ of the $j$th sample and $y_{j}$ denotes the class label of this sample (+1 for ASPD and -1 for controls). The Kendall tau correlation coefficient of the functional connectivity feature $i$ can be defined as:

$\tau_{i}=\frac{n_{c}-n_{d}}{m\times n}$ (1)

Where $n_{c}$and $n_{d}$ are the number of concordant and discordant pairs, respectively. Because we don’t consider the relationship of two samples, the total number of sample pairs is $m\times n$. For a pair of observation datasets $\left\{x_{ij},y_{j}\right\}$ and$\left\{x_{ik},y_{k}\right\}$, it is a concordant when

$\mathrm{sgn}(x_{ij}-x_{ik})=\mathrm{sgn}(y_{j}-y_{k})$ (2)

Correspondingly, it is a discordant pair when

$\mathrm{sgn}(x_{ij}-x_{ik})=-\mathrm{sgn}(y_{j}-y_{k})$ (3)

Thus, the positive correlation coefficient $\tau_{i}$ represents the $i$th functional connectivity that exhibits a significant decrease in the ASPD group compared with the control group, while the negative correlation coefficient$\tau_{i}$, represents the $i$th functional connectivity that exhibits a significant increase in the ASPD group compared with the control group. Moreover, this difference increases substantially when the absolute value of the Kendall correlation coefficient$\tau$is larger.
