# Supplementary material for: Identifying Individuals with Antisocial Personality Disorder Using Resting-State fMRI
Source: PLoS One. 2013 Apr 12;8(4):e60652. doi: 10.1371/journal.pone.0060652 (PMC3625191; doi:10.1371/journal.pone.0060652)
Supplement: Text S3 — A Voxel-based Morphometry Analysis and Results. (DOC) [file pone.0060652.s005.doc]

**Text S3: A Voxel-based Morphometry Analysis and Results**

A Voxel-based Morphometry (VBM) analysis was performed using the DARTEL in SPM8 ([http://www](http://www/). fil.ion.ucl.ac.uk/spm) and MATLAB 7.8 (Math-Works, Natick, MA, USA). This method is more deformable and notably improves the realignment of small inner structures to attain the best results. Structural images of each subject were checked to ensure that the images were in a suitable format. The “modulated” warped grey and white matter images were smoothed with a Gaussian kernel (full-width half maximum=8mm) to increase the signal-to-noise ratio. The spatially normalized images were specified 1.5 mm voxel sizes. After spatial pre-processing, the smoothed, modulated, normalised grey matter (GM) and white matter (WM) datasets were used for statistical analysis.

Statistical tests which involved locating regionally specific differences in gray matter and white matter between two groups were performed with the general linear model (GLM) implemented in SPM8. To avoid possible edge effects between different tissue types, we excluded all voxels with the GM or WM value of 0.2 (absolute threshold masking). Statistical parametric GM and WM maps were thresholded with p ≤0.001 uncorrected for multiple comparisons. The minimal number of contiguous voxels was set at 70 across the entire brain.

The areas with higher density in ASPD vs. control brains were detected with SPM8. Significant differences in GM and WM intensities between the two groups were found (Table S1). The ASPD group showed a significant volume increase in GM (Figure S1A) and WM (Figure S1B). Intensities in GM and WM regions did not show any significant reduction in ASPD compared with controls. These effects did not interact with gender or age.
